# Characterization of the Expression and Functions of Two Odorant-Binding Proteins of *Sitophilus zeamais* Motschulsky (Coleoptera: Curculionoidea)

**DOI:** 10.3390/insects10110409

**Published:** 2019-11-15

**Authors:** Ying Zhang, Chen Shen, Daosong Xia, Jian Wang, Qingfeng Tang

**Affiliations:** 1 Key Laboratory of Biology and Sustainable Management of Plant Diseases and Pests of Anhui Higher Education Institutes, College of Plant Protection, Anhui Agricultural University, Hefei 230036, Anhui, China; zhangying60416@163.com (Y.Z.); shenchen2506@163.com (C.S.); xiadaosong_25@163.com (D.X.); 2 Department of Entomology, University of Maryland, College Park, MD 20742, USA; jianwang@umd.edu

**Keywords:** *Sitophilus zeamais*, odorant-binding protein, expression pattern, host volatiles, ligand-binding spectrum, RNA interference, behavioral responses

## Abstract

Odorant-binding proteins (OBPs) are important in insect chemical communication. The objective of this research was to identify the functions of two OBPs in *Sitophilus zeamais*. qRT-PCR and western blot (WB) were performed to investigate the expression profiles at the transcript and protein levels, respectively. Fluorescence competitive binding assays were used to measure the ability of the OBPs to bind to host volatiles, and a Y-tube olfactometer was used to verify the results (attraction/no response) via behavioral experiments. The RNAi was used to verify the function by knocking down the ability of proteins to bind odorants. qRT-PCR showed the highest expression SzeaOBP1 and SzeaOBP28 at the low-instar larva (LL) and eclosion adult (EA) stages, respectively. WB showed that both SzeaOBP1 and SzeaOBP28 were highly expressed in the EA stage. Fluorescence competitive binding assays indicated that SzeaOBP1 exhibited extremely high binding affinity with cetanol. SzeaOBP28 exhibited a pronounced binding affinity for 4-hydroxy-3-methoxybenzaldehyde. The behavioral experiment showed that the adult *S. zeamais* responded strongly to 4-hydroxy-3-methoxybenzaldehyde and valeraldehyde from *Sorghum bicolor*. The RNAi knockdown individuals displayed behavioral differences between normal insects and dsRNA (SzeaOBP1)-treated insects. We infer that they both have functions in perception and recognition of host volatiles, whereas SzeaOBP28 may also have other functions.

## 1. Introduction

Insect olfaction has been a longstanding subject of scientific research aimed at understanding and identifying methods for controlling pest insects [1,2,3,4]. Detection of chemical signals from the environment via olfaction is an indispensable mechanism for the maintenance of an insect’s life, inducing critical behavioural responses at vital times. Insects can detect host or food volatiles in relatively complex environments, which plays an important role in the process of food localization by insects via the detection of plant volatiles. An understanding of the biological phenomena involved in the whole process of olfactory signal transduction in insects will not only aid the comprehension of host identification and other life activities of insects, but also facilitate the development of new and improved plant protection strategies [5].

Several researchers have investigated the whole olfactory responses of insects and the underlying mechanisms of olfaction by using odorant-binding proteins (OBPs), which will further the understanding of insect life [6,7,8,9]. However, relatively little is known regarding the maize weevil OBPs. On the basis of the characteristics of molecular biology, the specific functions of SzeaOBPs remain unknown or unclear. The associations between SzeaOBPs and the perception of host plant odorants and food resource volatiles in maize weevil are of great interest. The OBPs in the insect sensillum lymph play important physiological roles in both insect chemical communication and in survival [4]. OBPs are present as water-soluble proteins in olfactory sensilla. These proteins bind odorant molecules in the sensillum lymph and transport these molecules to olfactory receptors in dendritic membranes that then generate, transmit, and integrate electrical signals, which ultimately guide the insect’s response to changes in the external environment. The binding of OBPs to lipid-soluble odorant molecules is a foundational aspect of the communication between insects and their surroundings [10]. Stored-product insects, including the maize weevil, *Sitophilus zeamais* Motschulsky (Coleoptera: Curculionoidea), are serious pests that affect many agricultural commodities, causing severe postharvest loss via the effects of both live insects and metabolites, with values as high as 9% in developed countries to 20% or more in developing countries [11,12]. In conventional control methods, fumigants are generally used to control storage pests in combination with, on the basis of our analysis, insect detoxification enzyme-related genes such as cytochrome P450 (CYP450) and Glutathione S-transferase (GST) [13]. In addition, we searched for ways to use OBPs to control storage pests. In this study, to elucidate the function of SzeaOBP1, chemical ecological techniques and molecular biology were used to investigate this OBP, which was achieved by quantitative real-time PCR of different developmental stages and by comparison of transcriptomic data showing the expression levels in different tissues. Then, expression and purification of target proteins was performed by using a bacterial expression system. A fluorescence competitive binding assay was used to measure the binding of insect proteins to host plant volatiles. RNA interference was used to verify the results.

This study identified the putative functions of SzeaOBP1 and SzeaOBP28 in maize weevil, examined the molecular activity of these proteins and the behavioral responses in maize weevil, and assessed the potential functional application of these proteins for binding or attraction.

## 2. Materials and Methods

### 2.1. General Odorants

The odorants used in this study were chosen from food source volatiles of maize weevil, including host plant seed or grain volatiles. In total, 27 odorants were selected for use in the tests after surveying a sufficient number of literature reports and a large number of preliminary experimental results. All the odorant samples were sourced from Adamas-beta (Shanghai, China), Aladdin (Shanghai, China), or Tokyo Chemical Industry (Tokyo, Japan) at the highest purity available (Table 1).

### 2.2. Insect Rearing

The population of maize weevil used in this experiment from a laboratory colony maintained and reared in Entomology Ecology Laboratory, School of Plant Protection, Anhui Agriculture University (Hefei, Anhui, China). The laboratory colony originated from field collection, identification, and culture in the laboratory for more than five generations. The *S. zeamais* insects were reared in glass containers that were 9 cm in diameter 10 cm in height. The containers were covered with a plastic cap with a breathable copper mesh in the center and kept at 28 ± 1 °C under 80% relative humidity in total darkness. The experimental population samples were all done by random collection.

### 2.3. RNA Extraction and cDNA Synthesis

Total RNA extraction was performed for each sample using RNAiso Plus (TaKaRa, Dalian, China) according to the manufacturer’s instructions. The integrity and purity of the total RNA were analyzed by 1.5% agarose electrophoresis and a NanoDrop spectrophotometer (Thermo Fisher Scientific, Wilmington, DE, USA). Then, each RNA sample was reverse-transcribed to cDNA using a two-step method with the PrimeScript RT Reagent Kit with gDNA Eraser (TaKaRa, Dalian, China). The first step involved the removal of genomic DNA using 5× gDNA Eraser buffer (2.0 μL), gDNA Eraser (1.0 μL), total RNA (2.0 μL), and RNase-free dH_2_O up to 10 μL; the mixture was incubated at 42 °C for 2 min. Next, reverse transcription was performed to synthesize the first-strand cDNA with the following reagents: step 1 reaction solution (10.0 μL), PrimeScript RT enzyme mix I (1.0 μL), RT primer mix (4.0 μL), 5× PrimeScript buffer 2 (4.0 μL), and RNase-free dH_2_O (1.0 μL); the total volume of the system was 20 μL. Lastly, we incubated the reaction system at 37 °C for 15 min, followed by incubation at 85 °C for 5 s.

### 2.4. Quantitative Real-Time PCR (qRT-PCR)

qRT-PCR was conducted on a Bio-Rad CFX96 real-time system (Bio-Rad Laboratories, Hercules, CA, USA) using SYBR Premix Ex Taq II (Tli RNase Plus) in Hard-Shell 96-well PCR plates (HSP9655, Bio-Rad, Bio-Rad Laboratories, Hercules, CA, USA) covered with Microseal ‘B’ adhesive seals (MSB1001). Beta-actin was used as an endogenous control to normalize the expression of target genes and to correct for sample-to-sample variation. Gene-specific primers were designed using Primer-BLAST (https://www.ncbi.nlm.nih.gov/tools/primer-blast) for qRT-PCR and are listed in Table 2. OligoCalc (oligonucleotide properties calculator; http://biotools.nubic.northwestern.edu/Oligo Calc.html) was used to analyze the properties of all the primers in the experiment. The amplification efficiencies of the target and reference genes were assessed using gradient dilution templates [30]. qRT-PCR was performed in 25 μL reactions under the following two-step PCR amplification conditions (standard procedure): denaturation at 95 °C for 30 s, followed by 40 cycles of 95 °C for 10 s and 60 °C for 30 s. Finally, melting curve analysis was performed. To test the reproducibility of the data, three biological replicates and three technical replicates were examined. The negative controls were treated with ddH_2_O instead of DNA for the non-template reaction. Relative expression levels were determined using the comparative 2^−ΔΔCt^ method for relative quantification [31]. The significant differences between samples were determined by DPS (data processing system) software v9.5 with one-way analysis of variance (ANOVA) and Tukey’s post-hoc test [32]. The level of significance was set at *p* < 0.05. All experimental results were presented using GraphPad Prism 5.

### 2.5. Sequence Analysis and Phylogenetic Tree Construction

Sequence information for these two SzeaOBPs was obtained previously from the *S. zeamais* transcriptome database with National Center for Biotechnology Information (NCBI) accession numbers MK341130 and MK341157. Signal peptides were predicted using Signal P (http://www.cbs.dtu.dk/services/Signal P/) [33]. Multiple sequence alignment was performed using DNAMAN software. OBP sequences were searched against the NCBI database by running the BLAST program. The results were downloaded as comparable fasta files. Then, on the basis of the sequences, we aligned the OBP family sequences and chose the typical order in insects for comparison by the maximum likelihood (ML) method with 1000 bootstrap replicates in MEGA 7.0 software [34].

### 2.6. Cloning of SzeaOBPs and Construction of the Expression Vector

Sequence information for these two SzeaOBPs were obtained previously from the *S. zeamais* transcriptome database with NCBI accession numbers MK341130 and MK341157. Gene-specific primers were designed to clone the genes but remove the signal peptide sequences. Both clones were designed to include the BamH1 and Xhol restriction sites. SzeaOBP1 was cloned into the pET-28a expression vector, and SzeaOBP28 was cloned into the pET-32a expression vector. The PCR protocol was as follows: initial denaturation at 98 °C for 30 s, followed by 40 cycles of 98 °C for 10 s, 58 °C for 15 s (pET-SzeaOBP1) or 57 °C for 15s (pET-SzeaOBP28), and 72 °C for 30 s, and a final extension at 72 °C for 5 min. Samples were stored at 4 °C until use. The PCR products and expression vectors were separately digested with the same restriction enzymes—BamH1 and Xhol—to complete the double enzymatic digestion. The 50 μL digestion reactions were prepared as follows: PCR product or expression vector (30 μL), FastDigest Green buffer (5 μL), enzyme 1 (2 μL), enzyme 2 (2 μL), ddH_2_O (11 μL). The reaction solution was mixed evenly after gentle centrifugation and kept at 37 °C for 2–4 h. The target gene fragment was inserted into the expression vector to construct a recombinant prokaryotic expression vector containing the target gene sequence. Ligation was performed with T4 DNA ligase (Thermo Fisher Scientific, Waltham, MA, USA) in 25 μL reactions, which were kept at 22 °C for 2–4 h for completion of the reaction. The amplification products were purified by gel recovery with the MiniBEST Agarose Gel DNA Extraction Kit Ver.4.0 (TaKaRa, Dalian, China). Positive clones were sequenced by General Biosystems (Anhui, China). After sequencing inoculate 3 mL bacterial solution to 300 mL LB medium containing corresponding antibiotics, the correct bacterial clones were cultured in large volumes, and recombinant plasmid extraction was carried out using a plasmid extraction kit (Axygen, Union City, CA, USA). Finally, the plasmids were transformed into *Escherichia coli* (*E. coli*) BL21 derivative Rosetta (DE3) competent cells.

### 2.7. Prokaryotic Expression and Purification of the Recombinant pET-SzeaOBP Proteins

Expression of the recombinant SzeaOBP proteins was induced. First, expression was induced in a small culture. Two single colonies harboring pET-SzeaOBP1 or pET-SzeaOBP28 were selected and inoculated in 5 mL of LB liquid medium (cells containing pET-SzeaOBP1 were cultivated in medium containing 100 μg/mL ampicillin, and cells containing pET-SzeaOBP28 were cultivated in medium containing 50 μg/mL kanamycin sulfate) (Solarbio, Beijing, China); the cells were cultured overnight with shaking at 37 °C. Expression was induced in the experimental group by addition of isopropyl-beta-D-thiogalactopryranoside (IPTG) (Solarbio, Beijing, China) at a final concentration of 1 mM, and the cells were grown at 37 °C for an additional 4 h; expression was not induced in the control cells. The samples were centrifuged at 12,000 rpm for 10 min, and the supernatant was discarded. The bacterial cell pellet was lysed as follows: 20–30 μL of 5× loading buffer was added, and the suspensions were boiled for 10 min. Then, 10 μL samples were obtained for sodium dodecyl sulfate polyacrylamide gel electrophoresis (SDS-PAGE) analysis. Then, large-scale induction was performed. A total of 3 mL of bacterial culture was used to inoculate 300 mL of LB medium containing the corresponding antibiotics, and induction of expression was conducted in large quantities. The cells were lysed by ultrasonication as follows: the induced 300 mL cultures were centrifuged at 5000 rpm for 10 min, and bacterial pellets were obtained (approximately 1.2~2 g (wet weight) of bacteria was obtained by centrifugation of each 300 mL bacterial culture). The bacteria were resuspended in 10 mL of 0.01 M phosphate buffer saline (PBS) (pH = 7.4). Then, the bacteria were ultrasonicated at 100 W in an ice bath for 16 min and kept at 4 °C. Following from this, the cells were centrifuged at 5000 rpm for 10 min to obtain the precipitate and supernatant, and 20 μL of the supernatant sample was subjected to SDS-PAGE. The precipitate was resuspended in an 8 M urea solution containing of 10 mL of imidazole and ultrasonicated at 100 W for 10 min in an ice-water bath, and 20 μL of the sample was retained for electrophoresis. The samples were centrifuged at 5000 rpm and 4 °C for 15 min, and the supernatant was collected. Purification of the His-tagged proteins was performed by affinity column chromatography using Ni-NTA resin. The purification buffer used contained urea (8 M) and PBS (pH 7.8). The eluents were 30 and 300 mM imidazole in PBS (pH 7.8). First, the recombinant protein present in the supernatant was purified by Ni column chromatography. The eluent was placed in a dialysis bag, and dialysis was carried out with 1× PBS for completion of the purification of the supernatant.

Next, the recombinant protein present in the precipitate was purified by Ni column chromatography and placed in a dialysis bag. The protein was renatured by applying a urea buffer gradient (6, 4, 3, 2, 1, and 0 M) for 2 h at 4 °C. Dialysis was performed for completion of the purification of the precipitate. The dialysis buffer was formulated as follows: 5% glycerol, 1% L-arginine, 2% glycine, and urea, dissolved in 1× PBS solution. Finally, the recombinant protein samples were quantified as previously described.

### 2.8. Preparation of Multi-Clonal Antibody and Western Blot Analysis

New Zealand white rabbits were immunized four times with the recombinant purified protein. The whole blood was collected and detected by ELISA. The results were qualified and the antibodies were stored at −80 °C for reserve. All the samples were prepared and quantified using the Total Protein Extraction Kit (Thermo, Waltham, MA, USA) and then quantified using the BCA Protein Assay Kit (Thermo, Waltham, MA, USA). SDS-PAGE analysis was performed with an 8%–12% separating gel and 5% concentrating gel, with 60 μg of total protein sample per pore at 10–15 μL per pore. The concentrating gel was run at 60 V, and the separating gel was run at 80 V; electrophoresis was performed for approximately 2 h. A PVDF membrane (Millipore, Shanghai, China) was soaked in methanol for 20 s and then transferred to Tris-glycine transfer buffer (containing 5% methanol) for at least 5 min. The SDS-PAGE gel was equilibrated for at least 30 min in Tris-glycine transfer buffer. Under cooling conditions, 100 V was applied to transfer the proteins to the membrane for 2 h at constant voltage and constant humidity. Then, the transfer film was sealed. After the transfer process was completed, the transfer film was placed in T-TBS (containing 5% skimmed milk powder or BSA). The transfer film was sealed at room temperature for 1 h and then rinsed three times with T-TBS for 5 min. Primary antibody (Huabio, Hangzhou, China) and secondary antibody hybridization followed by primary antibody blocking were performed in T-TBS (containing 3% skimmed milk powder or BSA) at a specific proportion, and the transfer film was incubated overnight at 4 °C. Then, the transfer film was rinsed four times in T-TBS for 5 min. The secondary antibody was dissolved in T-TBS (containing 2% skimmed milk powder) and incubated with the transfer film at room temperature for 1 h. Then, the transfer film was rinsed five times in T-TBS for 5 min. Afterwards, for signal detection, SuperSignal West Dura Extended Duration Substrate (Thermo Pierce, Wilmington, DE, USA) was used to prepare 1 mL of ECL (GE, Fairfield, CT, USA) working fluid according to the manufacturer’s instructions. The transfer film was incubated with the working fluid at room temperature for 1 min, and then the excess ECL reagent was removed, and the transfer film was sealed with X-ray film (Hua Dong Medicine, Shanghai, China) in a dark box for exposure for 5–10 min before fixing and developing.

### 2.9. Fluorescence Competitive Binding Assays

Emission fluorescence spectra were recorded on a Fluoromax-4500 fluorescence spectrophotometer (Hitachi, Tokyo, Japan) at room temperature in right-angle configuration, with a 1 cm light-path quartz cuvette and 5 nm slits for both excitation and emission. The protein was dissolved in 50 mM Tris-HCl buffer (pH 7.4), and ligands were added as 1 mM methanol solutions. A methanol solution of N-Phenyl-1-naphthylamine (1-NPN) (1 mM) was used as the fluorescent probe, and chromatography-grade methanol was used as the solvent. For determination of the binding constants of the SzeaOBPs with the probe, the excitation wavelength was set at 337 nm, and the emission wavelength was increased from 370 to 500 nm for scanning. The target SzeaOBP proteins were diluted in Tris-HCl buffer (pH 7.4) to a final concentration of 2 μM. After the sample was added, the fluorescence intensity was allowed to stabilize (approximately 15 s), and the maximum fluorescence intensity was recorded; the binding constant between the protein and probe was determined. This process was repeated three times for each sample. Then, the ligands (odorant standard samples) were diluted with pure methanol for competitive binding analysis. The final concentration of each ligand was 1 M. The final concentration of ligands in the colorimetric dish increased by 2 μM upon adding the ligands one by one. The fluorescence intensity was recorded from 2 to 16 μM. The binding constants between the target SzeaOBP proteins and the ligands were determined. The fluorescence intensity was measured by adding 50 mM Tris-HCl buffer, SzeaOBP protein, and the fluorescence probe 1-NPN to the colorimetric dish for fluorescence measurement. The final concentration was 2 μM. When the fluorescence intensity was stable, the maximum fluorescence intensity was recorded, and then the ligands dissolved in methanol were added to the colorimetric dish one by one. The concentration increased from 2 μM, and the changes in fluorescence intensity were recorded. Each measurement was repeated three times.

To calculate the binding constants, the fluorescence intensity and ligand concentration were plotted using GraphPad Prism 5. Assuming that 1-NPN binds to the protein at a 1:1 ratio and that the protein was 100% active, the curve was linearized using a Scatchard plot. IC_50_ (ligand concentration at which the fluorescence intensity of the complex decreases by half) values were used to calculate the dissociation constants of the SzeaOBPs and the ligands by the following equation: Ki = [IC_50_]/(1+[1-NPN]/K_1-NPN_), where [1-NPN] is the concentration of the unconjugated ligand, and K_1-NPN_ represents the dissociation constants of the SzeaOBPs and 1-NPN.

### 2.10. Behavioural Experiments Using a Y-Tube Olfactometer

The behavioral experiments were designed to test the results for selected odorants. The odorant compounds used in behavioral experiments are from Table 1. To avoid the effects of temperature and light on the experiment, the evaluations were performed in a constant temperature and humidity (CTH) room at 28 ± 1 °C and 80% relative humidity under dim light supplied by a red fluorescent tube with no natural lighting. A Y-tube olfactometer system that was previously described by Yusuf and Takabayashi [35,36] was used to test the attractiveness of different odorant treatments to adult maize weevils. The system comprised a central tube (10 cm long) and two lateral arms (each 15 cm long and 15 mm wide). The angles between the two arms were 75°. Each arm was connected to a glass chamber (200 mL capacity) holding the different odorants. The different parts of the device were connected in the following order: air pump, activated carbon filter, distilled water humidifier, odorant source device, gas flow meter, and Y-tube olfactometer. The parts were connected by Teflon tubes. Airflow through both arms was generated by applying air pressure, and the air was purified using a charcoal-filled gas wash bottle. The airflow through the arms of the Y-tube (150 mL/min/arm) was adjusted by using flow metres, which were positioned between the flasks and the Y-tube. A clean Y-tube was used for each test to avoid carryover of odorants.

Before bioassays, according to the experimental requirements, the odorant source was placed in the odorant source device, which was then ventilated for 15 min so that the odorant filled the tube to ensure accuracy of the test results. The antennae and appendages of the selected insects were verified to be intact, and the insects were confirmed to be active. The insects were introduced from the base of the main arm of the Y-tube olfactometer. The selected odorant was used for the treatment group, and a blank was used for the control group. A single adult *S. zeamais* insect was introduced into the Y-tube olfactometer at the entrance of the stem, with a maximum observation duration of 10 min per beetle, and the responses were considered positive when the insects reached at least 2 cm along the arm connected to the test chambers. The lack of a positive response was recorded if an insect did not reach 2 cm after 10 min of observation. Insects were used only once and then discarded. Each odorant was used to test 30 adult maize weevils, and the number of male and female insects per test was the same. Three groups of experiments were repeated for three times. When calculating results, only the number of insects making the selection was counted, and the insects that did not made a selection were not included.

### 2.11. RNA Interference

Specific primers for SzeaOBP1 were designed to amplify the fragment to synthesize the double strand RNA. The primers were used by adding the T7 sequence polymerase binding during amplification. The primers were as follows: dsSzeaOBP1-F: TAATACGACTCACTATAGGGGGAACAAACGACCCCCAA; dsSzeaOBP1-R: TAATACGACTCACTATAGGGAAGATTGTGGTCCTCTTTAA. dsGFP-F: TAATACGACTCACTATAGGGAGTGCTTCAGCCGCTACCC; dsGFP-R: TAATACGACTCACTATAGGGGCGCTTCTCGTTGGGGTC. The primers were chosen according to different regions in the qRT-PCR test. The green fluorescent protein was used to compare the control and the negative control (GenBank accession number: KF410615.1). The double-stranded RNA (dsRNA) was synthesized using an HTE Thermo Scientific following the manufacturer’s instructions. The purity and integrity of dsRNA was confirmed by 1% agarose gel electrophoresis. The dsRNA concentration was determined by NanoDrop spectrophotometer (Thermo Fisher Scientific, USA), and stored in the RNase-free tube at −80 °C until used.

The treatment was fed to the adult insect every 12 h, and we collected the insect after 72 h treatment. Three treatments were set up for RNA interference (RNAi) consisting of RNase-free water (controls), dsGFP, and dsRNA group. Thirty *S. zeamais* adults in each group were used for the interference assays with the dose of 3 μg, and were repeated three times. The efficiency of the RNA interference was verified using qRT-PCR by extracting the total RNA from each group. The behavior responses of different treatments were assessed in a Y-tube olfactometer.

## 3. Results

### 3.1. Comparison of Relative mRNA Expression

**Figure 1:**
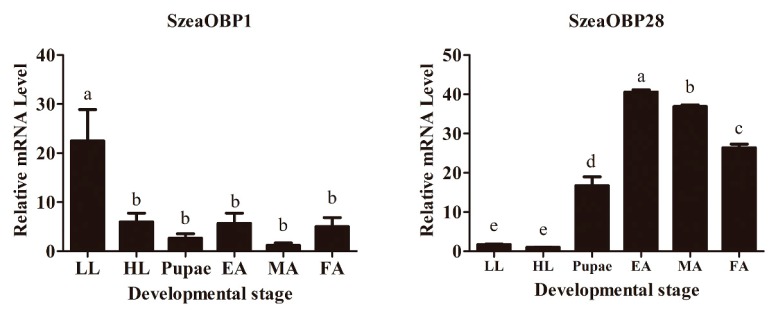
Relative mRNA levels. Expression patterns of various *Sitophilus zeamais* odorant-binding protein (OBP) genes in different developmental stages, including LL (low-instar larva), HL (high-instar larva), pupae (prepupa and postpupa), EA (eclosion adult), MA (male adult), and FA (female adult). The gene expression levels in the various tissues were normalized relative to the expression in different tissues. The data are presented as the means of three replicates (*n* = 3) ± SE. Different lowercase letters indicate significant differences (*p* < 0.05).

Total RNA samples of high quality and purity were obtained, and cDNA, which was obtained by reverse transcription, was used as the template for qRT-PCR. Insects at six different developmental stages, including LL (low-instar larva), HL (high-instar larva), pupae (prepupa and postpupa), EA (eclosion adult), MA (male adult), and FA (female adult) exhibited different levels of SzeaOBP1 and SzeaOBP28, as shown in Figure 1. The SzeaOBP1 levels in the LL samples were statistically significant. The expression level of SzeaOBP1 was highest in the LL stage. The next highest levels were observed for the HL, EA, and FA stages. Compared with other stages, the expression levels in the pupae and MA were the lowest. This finding may indicate that the SzeaOBP1 levels are highest during the early larval stage, decreasing gradually with development. The expression of SzeaOBP28 was highest in the EA, MA, and FA stages, followed by pupae, and the lowest levels were observed in the LL and ML stages. In general, the expression level was low at the early stage of development and high at the EA and adult stages.

### 3.2. SzeaOBP Sequence Alignment and Phylogenetic Analysis

**Figure 2:**
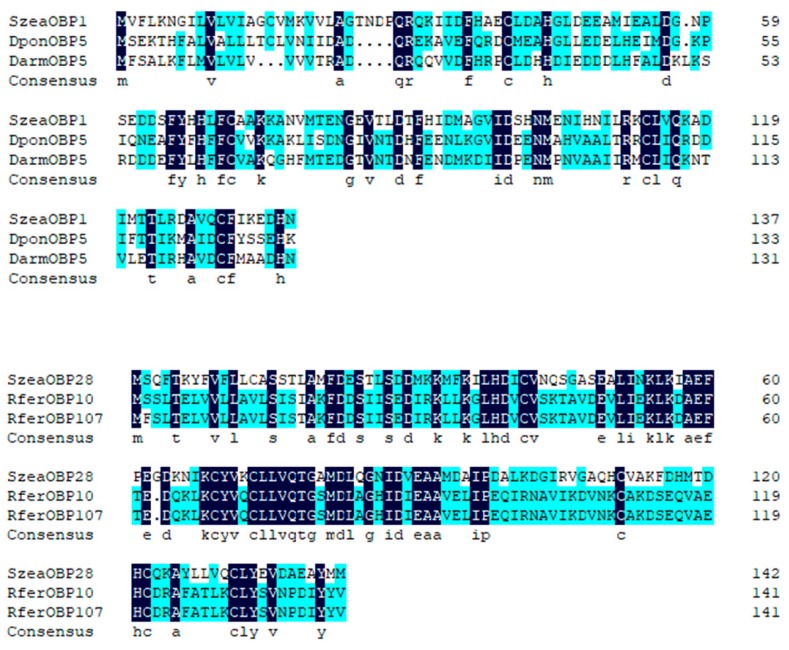
Multiple sequence alignment of OBPs from *Sitophilus zeamais* and other Coleoptera insects. The species names and GenBank accession numbers of the four OBPs are as follows: *Dendroctonus ponderosae* (DponOBP5, AKK25133.1); *Dendroctonus armandi* (DarmOBP5, ALM64967.1); *Rhynchophorus ferrugineus* (RferOBP10, ANE37554.1; RferOBP107, AVR54529.1).

**Figure 3:**
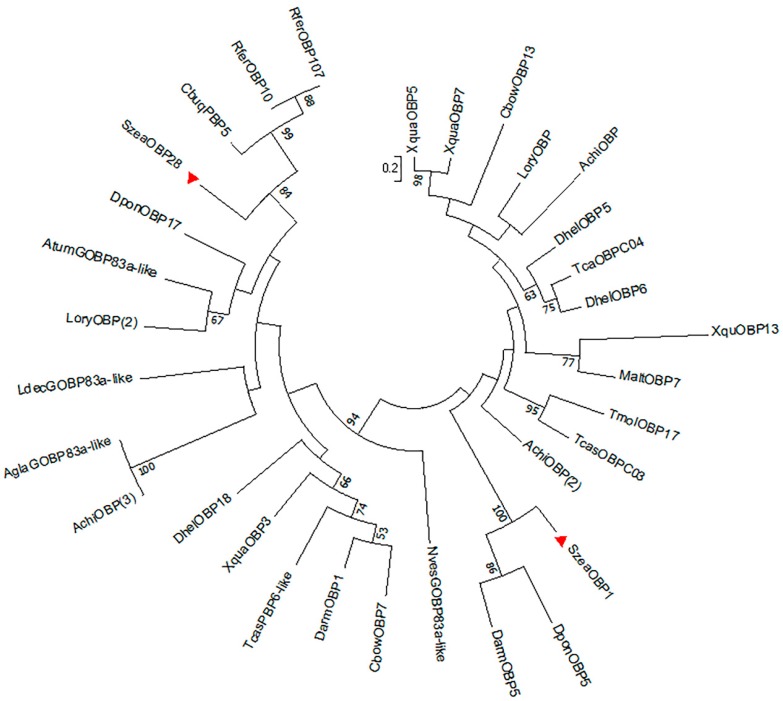
Maximum likelihood tree of 32 OBPs from *Sitophilus zeamais* and other Coleopteran insects with 1000 bootstrap replicates. The species names and GenBank accession numbers for all the OBP sequences as follows: *Aethina tumida* (AtumGOBP83a-like, XP_019868892.1); *Anoplophora chinensis* (AchiOBP, AUF72980.1; AchiOBP, AUF72977.1; AchiOBP, AUF72991.1); *Anoplophora glabripennis* (AglaGOBP83a-like, XP_023310142.1); *Colaphellus bowringi* (CbowOBP7, ALR72495.1; CbowOBP13, ALR72501.1); *Cyrtotrachelus buqueti* (CbuqPBP5, APG79366.1); *Dastarcus helophoroides* (DhelOBP5, AIX97051.1; DhelOBP6, AIX97052.1; DhelOBP18, AIX97064.1); *Dendroctonus armandi* (DarmOBP1, AIY61044.1; DarmOBP5, ALM64967.1); *Dendroctonus ponderosae* (DponOBP5, AKK25133.1; DponOBP17, AKK25141.1); *Leptinotarsa decemlineata* (LdecGOBP83a-like, XP_023019484.1); *Lissorhoptrus oryzophilus* (LoryOBP, AHE13799.1; LoryOBP, AHE13800.1); *Monochamus alternatus* (MaltOBP7, AIX97022.1); *Nicrophorus vespilloides* (NvesGOBP83a-like, XP_017781594.1); *Rhynchophorus ferrugineus* (RferOBP10, ANE37554.1; RferOBP107, AVR54529.1); *Tenebrio molitor* (TmolOBP17, AJM71491.1); *Tribolium castaneum* (TcasOBPC03, EFA07546.1; TcasOBPC04, EFA07430.1; TcasPBP6-like, XP_015835846.1); *Xylotrechus quadripes* (XquaOBP3, AXO78381.1; XquaOBP5, AXO78383.1; XquaOBP7, AXO78385.1; XquaOBP13, AXO78391.1).

The SzeaOBP1 sequence consisted of 417 bp, and the SzeaOBP28 sequences consisted of 432 bp, encoding 138 and 143 amino acids, respectively. The initial 23 and 19 amino acid residues of SzeaOBP1 and SzeaOBP28, respectively, were predicted to constitute the signal peptides. Sequence alignment of SzeaOBP1 with DponOBP5 and DarmOBP5 showed similar sequence identity (identity >55.40%), whereas sequence alignment of SzeaOBP28 with RferOBP10 and RferOBP107 had high homology (identity >79.72%). SzeaOBP1 had only four conserved cysteine residues, exhibiting loss of two conserved cysteines, suggesting that this protein belonged to the Minus-C OBP subfamily of proteins. Sequence alignment showed that SzeaOBP28 possessed a typical six-cysteine motif, that is, C_1_-X_27_-C_2_-X_3_-C_3_-X_37_-C_4_-X_10_-C_5_-X_8_-C_6_, where X is any amino acid. SzeaOBP28 belonged to the classic OBP subfamily of proteins (Figure 2). An ML phylogenetic tree was constructed using the sequences of SzeaOBP1, SzeaOBP28, and 30 OBPs from eight Coleoptera insects. Minus-C OBPs and classic OBPs formed two branches: Minus-C OBPs were clustered with SzeaOBP1, and classic OBPs were clustered with SzeaOBP28. SzeaOBP1 was clustered with DponOBP5, and DarmOBP5, SzeaOBP28, RferOBP10, and RferOBP107 were clustered together, indicating that these genes have slow evolution rates and are highly conserved, which is consistent with the results of multiple sequence alignment (Figure 3).

### 3.3. Cloning Results and Bacterial Expression

SzeaOBP1 and SzeaOBP28 were successfully amplified, and cohesive ends were generated by PCR for ligation with the expression vector. After verification of the fragments encoding the recombinant protein by sequencing, the target sequence, after removal of the signal peptide-encoding sequence, was successfully cloned into pET-28a for SzeaOBP1 and pET-32a for SzeaOBP28. The encoded SzeaOBP1 protein was His-tagged; the predicted molecular weight was approximately 19 kDa. The encoded SzeaOBP28 protein was His-tagged and Trx-tagged; the predicted molecular weight was approximately 32 kDa. SDS-PAGE was performed 4 h after induction with 1 mM IPTG at 37 °C, and distinct bands were observed at the expected size of 19 kDa. After purified by column chromatography, bands corresponding to SzeaOBP1 (at 19 kDa) were present in the supernatant and precipitate, which indicated that this protein was expressed in both the supernatant and inclusion bodies. Bands corresponding to SzeaOBP28 (32 kDa) were present in the precipitate, indicating that this protein was expressed in inclusion bodies.

**Figure 4:**
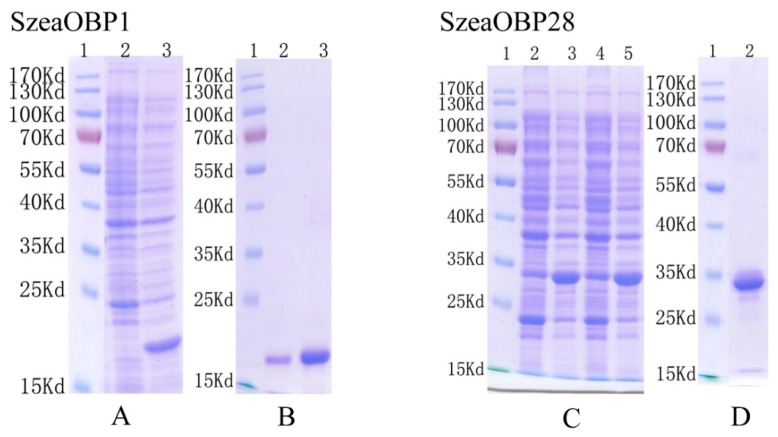
SDS-PAGE and western blot analysis of the expressed products and purified pET-SzeaOBPs. (**A,C**) show the pET-SzeaOBP recombinant protein that was primarily expressed by induction with isopropyl-beta-D-thiogalactopryranoside (IPTG) and detected by SDS-PAGE. (**B,D**) show western blot analyses of the purified SzeaOBPs. Lane 1 in all the images shows the protein molecular weight marker. (**A**) lane 2: SzeaOBP1-transformed *Escherichia coli*, not induced; lane 3: SzeaOBP1-transformed *Escherichia coli*, induced. (**B**) lane 2 and 3: recombinant purified SzeaOBP1 protein. (**C**) lanes 2 and 4: SzeaOBP28-transformed *Escherichia coli*, not induced; lanes 3 and 5: SzeaOBP28-transformed *Escherichia coli*, induced. (**D**) lane 2: recombinant purified SzeaOBP28 protein.

After elution with 30 and 300 mM imidazole, distinct bands for SzeaOBP1 were observed at the expected size of 19 kDa. After elution with 300 mM imidazole, SzeaOBP28 exhibited distinct bands at the expected size of 32 kDa. Protein dialysis and quantitative results showed that there were distinct bands corresponding to SzeaOBP1 at 19 kDa and SzeaOBP28 at 32 kDa (Figure 4). The isoelectric points of SzeaOBP1 and SzeaOBP28 were 4.95 and 4.92, respectively.

### 3.4. Antibody Production and Western Blot Analysis of the Two Recombinant Proteins

**Figure 5:**
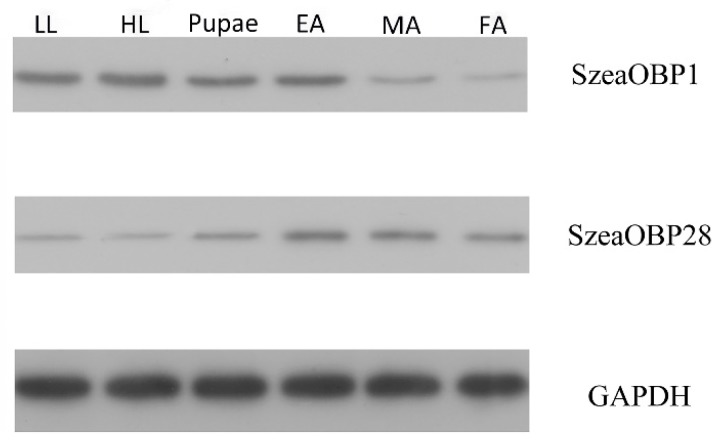
Protein expression patterns of the two proteins in different developmental stages. Detection of expression levels in different development stages of *Sitophilus zeamais*, including LL (low-instar larva, HL (high-instar larva), pupae (prepupa and postpupa), EA (eclosion adult), MA (male adult), and FA (female adult). The protein molecular weight marker is shown on the left. GAPDH was the control.

The effect of rabbit serum was evaluated and detected on the basis of OD values at different dilution ratios. The OD 450 nm of SzeaOBP1 at a ratio of 1:64,000 was 1.895, and the OD 450 nm of SzeaOBP28 at a ratio of 1:64,000 was 2.342. The values were all greater than 0.6, which was indicative of good quality. Total protein was extracted from *S. zeamais* at six different developmental stages. The total protein concentrations obtained for the LL, HL, pupae, EA, MA, and FA stages were 2108.3 μg/mL, 1705.6 μg/mL, 2486.8 μg/mL, 1605.38 μg/mL, 2258.5 μg/mL, and 1699.6 μg/mL, respectively. The western blot (WB) analysis showed that SzeaOBP1 was highly expressed at the LL, HL, pupae, and EA stages but expressed at low levels at the MA and FA stages. The expression level of SzeaOBP28 was highest at the EA and MA stages, followed by the pupae and FA stages, and the lowest levels were observed at the LL and HL stages (Figure 5).

### 3.5. Fluorescence Competitive Binding Assays

**Figure 6:**
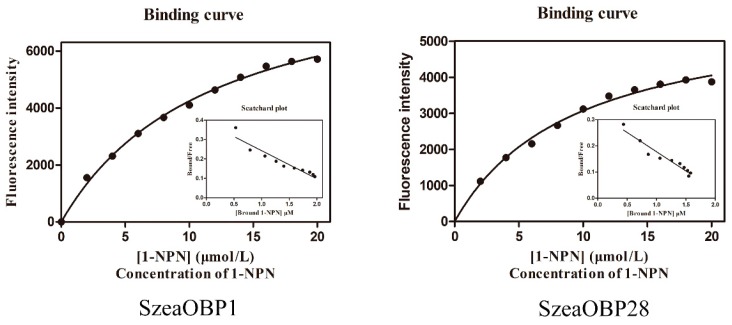
Binding curves and Scatchard plots of the fluorescence probe 1-NPN with SzeaOBP1 and SzeaOBP28. The binding curves and corresponding Scatchard plots indicate that the binding constant of the SzeaOBP1/1-NPN complex was 8.590 μM, and that of the SzeaOBP28/1-NPN complex was 7.214 μM.

Upon excitation at 337 nm, the intrinsic fluorescence intensity was low with only Tris buffer and 1-NPN added, with a value of only 337.16 ± 3.9. However, in the presence of recombinant proteins, the spectrum shifted from 470 nm to approximately 418 nm, accompanied by a sharp increase in fluorescence intensity. The fluorescence values for SzeaOBP1 and SzeaOBP28 reached 1938.33 ± 72 and 2511.33 ± 85, respectively. This finding indicates that the fluorescence probe 1-NPN is suitable for studying the binding of ligands to the two SzeaOBPs. The binding curve for the fluorescent probe 1-NPN with SzeaOBPs is shown in Figure 6.

**Figure 7:**
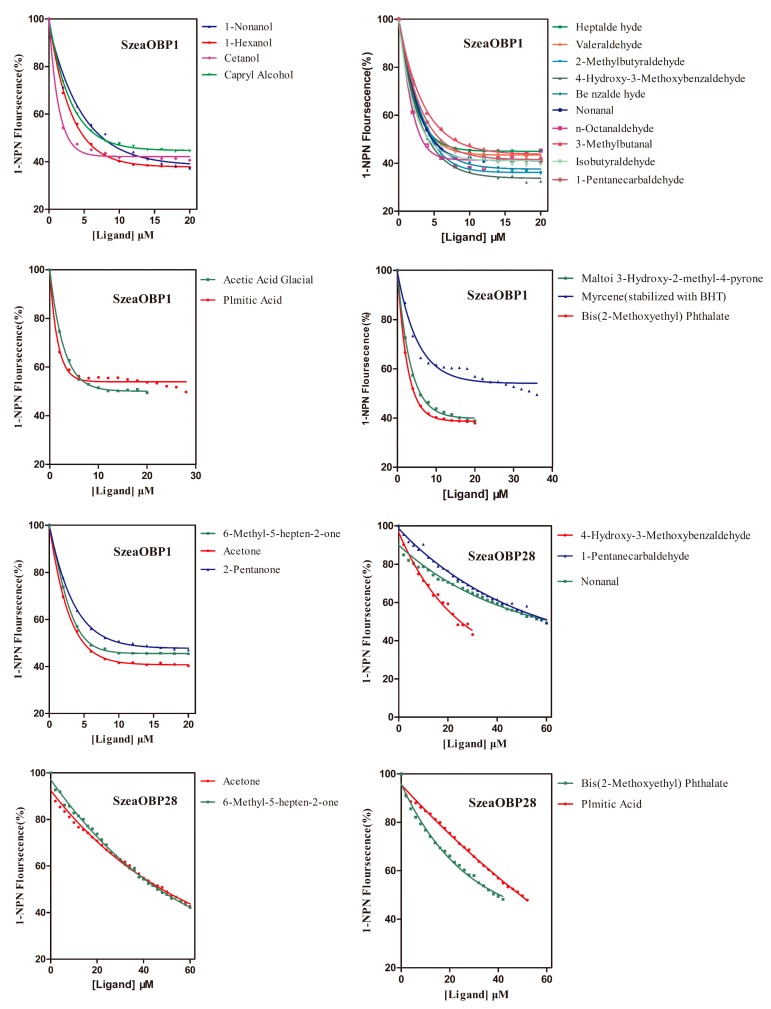
Fluorescence competitive binding curves of the two SzeaOBPs with host plant volatiles. The recombinant proteins and 1-NPN were diluted with 20 mM Tris-HCl buffer (pH 7.4) to a final concentration of 2 μM. The mixed solution was titrated with each host plant volatile (1 mM) to a final concentration of 0–60 μM.

The K_1-NPN_ dissociation constants of the SzeaOBPs complexes were 8.590 μM and 7.214 μM for SzeaOBP1 and SzeaOBP28, respectively. Then, the dissociation constants of the SzeaOBPs and ligands were calculated using K_1-NPN_ values. SzeaOBP1 was highly specific for the major components of all 27 ligands, exhibiting the strongest affinity for cetanol (2.748 ± 0.139 μM) and the weakest affinity for myrcene (30.27 ± 0.798 μM). SzeaOBP28 exhibited a specific binding spectrum with the minor ligands, showing pronounced binding affinity for 4-hydroxy-3-methoxybenzaldehyde (20.23 ± 1.918 μM). Both recombinant SzeaOBP proteins did not bind to decanal, linoleic acid, (+)-α-pinene, or the alkanes. The binding affinities of the 27 ligands to the SzeaOBPs are listed in Table 3 and shown in Figure 7.

### 3.6. Behavioural Response to Host Plant Volatile Components

**Figure 8:**
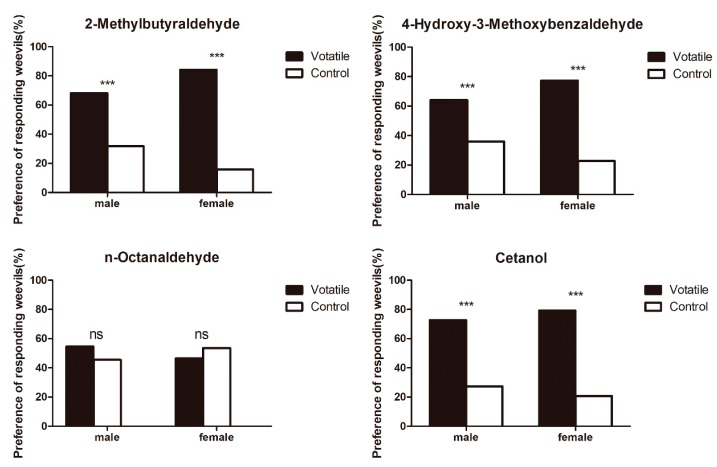
Responses of male and female *Sitophilus zeamais* adults to volatiles from four host plant combinations in the Y-tube olfactometer assays. Activity is expressed as percentage of weevils responding, and bars followed by different markers indicate significantly different activity of the weevils between the treatments (*p* < 0.05). The mean values shown are predicted values from regression analysis. ‘*’ indicates significant differences from even distribution at * *p* < 0.05, or ** *p* < 0.01, *** *p* < 0.001. ‘ns’ indicates not significantly different.

According to the experimental results in the previous section, four odorant compounds with strong ability were selected for behavioral experiments. The responses of *S. zeamais* to compounds with strong binding ability from different host plants were determined (Figure 8). Volatiles from the intact host plant grains were selected by comparison of the responses of male and female *S. zeamais* insects. Of the different standard odorant sample treatments, the 4-hydroxy-3-methoxybenzaldehyde treatment stimulated activity in a significantly higher number of normal male and female weevils than the control treatment. The active normal male and female weevils were strongly attracted to volatiles, with males exhibiting slightly stronger attraction than females. Significant differences in male and female weevils’ attraction to volatiles were also found between the 2-methylbutyraldehyde and the cetanol. There was only a slight difference in the response of females and males to the three odorant compounds. The stimulation of n-octanaldehyde had no obvious effect on the female and male of maize weevils.

### 3.7. Effect of dsRNA Treatment on Sitophilus Zeamais Behaviour

**Figure 9:**
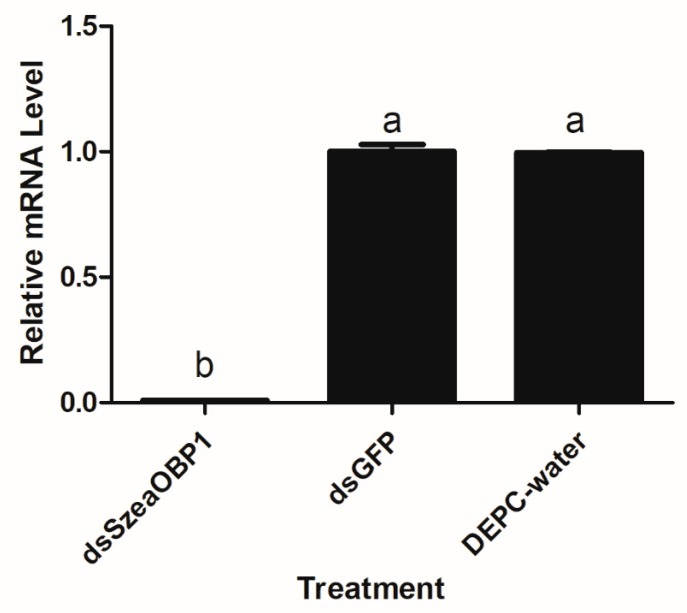
Effect of RNAi treatment on the transcript levels of SzeaOBP1 in adults. DEPC-water and GFP were compared as controls.

**Figure 10:**
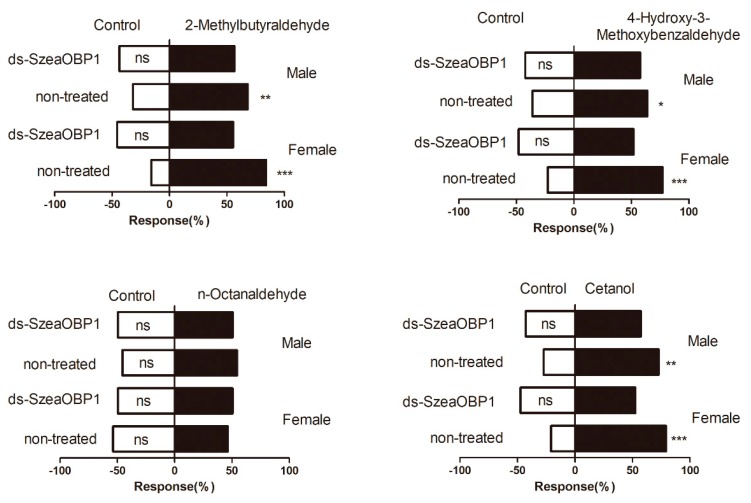
Behavioral responses of *Sitophilus zeamais* to four selected volatiles in a Y-tube olfactometer after dsRNA treatment. Activity is expressed as percentage weevils responding, and bars followed by different marker indicate significantly different activity of the weevils between the treatments (*p* < 0.05). The mean values shown are predicted values from regression analysis. ‘*’ indicates significant differences from even distribution at * *p* < 0.05, or ** *p* < 0.01, *** *p* < 0.001. ‘ns’ indicates not significantly different.

After the 72 h dsRNA feeding treatment with the adult *Sitophilus zeamais*, qRT-PCR was used to detect the relative reduction of transcript level. The expression was reduced by 80% with SzeaOBP1 in adults. The attraction to plant volatiles was tested using the Y-tube olfactometer with the same volatiles as before. The OBP1 treatment was significantly different compared with the feeding group. The dsRNA (SzeaOBP1)-fed adults were significantly affected in this experiment. Meanwhile, dsRNA (SzeaOBP1)-treated beetles showed lower attraction to these three volatiles than normal insects, indicating that SzeaOBP1 is important in perception of these odorants (Figure 9 and Figure 10).

## 4. Discussion

Researchers have used many classic methods for functional research on insect antennae, including the use of scanning electron microscopy to study the ultrastructures of antennal sensilla [37,38]. For odorant receptors, Wang and his team used systematic functional analysis across much of the conventional insect odorant receptor repertoire in *Xenopus oocytes* using two-electrode, voltage-clamp electrophysiology [39,40]. The purpose of this study was to explore the functions of OBPs in *S. zeamais*. We used typical methods of reverse chemical communication and molecular biology with volatiles from host plants to study the mutual correspondence between the insects and plants. The specific important roles that OBPs play in insect life were predicted, such as food localization. To truly achieve the purpose of functional research, this feature must be widely applied [41,42]. At the beginning of this study, our laboratory obtained the GenBank transcriptomic dataset from previous work [43]. We also identified 41 OBPs using qRT-PCR analysis and 64 odorant receptors, including odorant co-receptors, and studied these genes in different tissues, including male and female antennae [44]. To predict and conduct research on the specific functions of these proteins, two OBPs, namely, SzeaOBP1 and SzeaOBP28, were used in this study, and detailed analysis was performed. qRT-PCR was used to compare the different expression levels in six different developmental stages of *S. zeamais*. As expected, different expression levels were observed in the different developmental stages. SzeaOBP1 and SzeaOBP28 exhibited the highest expression levels in antennae compared with other insect tissues in our previous work [43]. Consistent with previously reported results, SzeaOBP1 expression level in FAs remained higher than that in Mas, and SzeaOBP28 expression level in MAs remained lower than those in FAs.

The larvae reared in the controlled closed environment used mostly intact grain as host material to live in, only damaging the grain by biting. In the adult stage, localization of food and selection of spawning sites becomes increasingly important. Prediction of the functional roles of OBPs in the whole olfactory system is important [45]. Therefore, focused research on this aspect is required to prevent grain loss during storage and for pest management. Gene expression ultimately leads to the production of corresponding proteins (or enzymes) [46]. According to the results of qRT-PCR, SzeaOBP1 may be involved in the identification of internal food by LL, and SzeaOBP28 may be involved in the related living conditions of LL and MAs, including food location and mating behavior. Therefore, detected proteins are important markers and indicators of gene expression.

On the basis of the expression results of SzeaOBPs on transcription and protein levels, it can be found that the expression trend of SzeaOBP28 at the level of transcription and protein was basically the same, whereas the expression trend of SzeaOBP1 at these two levels had some differences. Protein synthesis is very complicated, and protein degradation is also a common phenomenon. Even when mRNA expression levels remain high, at the protein level, different trends can be observed, which is a reasonable observation [47,48,49]. RNA levels and protein levels are often regulated independently. Many microarray/proteomic comparisons have demonstrated low correlation between expression levels or changes in expression levels between protein and RNA, which is a reasonable observation [50,51]. In fact, the results are very interesting and raise questions regarding the biochemical regulation of these specific RNAs and proteins.

Many methods can be used to study the functions of OBPs, but fluorescence competitive binding assays may be the most effective. The fluorescence competitive binding assays showed that each OBP protein binds to these volatiles from the host food source. The volatiles in intact grains are, to a certain extent, different from those in grains damaged by the pest. The odorants tested were distinct between the stored grain volatiles and the green leaf volatiles (GLVs); simultaneously, the differences in odorants between damaged grains and intact grains cannot be ignored. A Scatchard plot was used to examine the binding of the proteins to the odorants [52,53]. The behavior experiment performed with the Y-tube olfactometer simulated, on average, the actual behavior in a technical setting and the fact that the results reflect the actual situation after controlling for external influences. The movement of the insects can be accurately recorded, and the results are reliable. On the basis of the experimental results, the selectivity trend demonstrated by the behavioral experiments was consistent with the trends observed for most molecular biological experiments for a single odorant, as demonstrated by the results for the compounds 4-hydroxy-3-methoxybenzaldehyde. It is generally recognized that behavioral experiments most reliably reflect the actual situation and can be used to validate and reproduce the results of molecular experiments in a live system [54,55]. The results of this study, from the molecular experiments to the behavioral test, demonstrate the functional relationships between the two insect OBPs and the corresponding host plant volatiles in maize weevils. These findings may facilitate further in-depth research on boring pests. RNA interference was used as the verification method to verify the function; the results of behavioral experiments showed that the RNAi knockdown individuals displayed behavioral differences between normal insects and dsRNA (SzeaOBP1)-treated insects. With regard to the insect chemical sensory mechanism, the results of this study are not sufficient for elucidation of this mechanism. On the basis of these findings, this approach could certainly be extended to all SzeaOBPs.

Overall, our tests for the ligands and proteins demonstrate that SzeaOBP1 exhibits high specificity for the major component and strong binding of this component, except alkanes, to which this protein did not respond. SzeaOBP28 exhibits a lower binding ability than SzeaOBP1 but remains important for research purposes due to the expression of this protein from the EA to adult stages.

## 5. Conclusions

In conclusion, the relative expression level at transcript and protein of these two SzeaOBPs were tested, we found that they showed different in distinct development stages. The tests for the ligands and proteins demonstrate that SzeaOBP1 exhibits high specificity for the majority component and strong binding of these components, except alkanes, to which this protein did not respond. SzeaOBP28 exhibits a lower binding ability than SzeaOBP1 but remains important for research purposes due to the expression of this protein from the EA to adult stages. The RNAi knockdown individuals displayed behavioral differences between normal insects and dsRNA (SzeaOBP1)-treated insects. We infer that they both have functions in perception and recognition of host volatiles, whereas SzeaOBP28 may also have other functions.

## Figures and Tables

**Table 1:** Volatiles from host plants used for fluorescence competitive binding experiments, including reagent name, chemical abstracts service (CAS) number, purity, source, and reference. All the volatiles selected were single compounds that have been reported in the literature.

Ligand	CAS	Purity (%)	Source	Origin	Reference	Molecular Weight	Formula
Pentadecane	629-62-9	99	Aladdin	*S. bicolor*	[14]	212.41	C_15_H_32_
N-hexadecane	544-76-3	98+	Adamas-beta	*S. bicolor*	[15]	226.44	C_16_H_34_
1-Nonanol	143-08-8	98	Adamas-beta	*A. hypogaea*	[16,17]	144.25	C_9_H_20_O
1-Hexanol	111-27-3	98	Adamas-beta	*O. sativa*, *S. bicolor*, *S. italica*	[15,16,18]	102.17	C_6_H_14_O
Capryl alcohol	111-87-5	99	Adamas-beta	*S. bicolor*	[15]	130.23	C_8_H_18_O
Cetanol	36653-82-4	98+	Adamas-beta	Storage grain	[19]	242.44	C_16_H_34_O
Decanal	112-31-2	97	Adamas-beta	*S. bicolor*	[15]	156.27	C_10_H_20_O
Valeraldehyde	110-62-3	98	Adamas-beta	*S. bicolor*	[14,20]	86.13	C_5_H_10_O
Heptaldehyde	111-71-7	97	Adamas-beta	*O. sativa*	[21]	114.19	C_7_H_14_O
4-Hydroxy-3-methoxybenzaldehyde	121-33-5	99	Adamas-beta	*T. aestivum*, *O. sativa, A. ativa, S. cereale*, *H. vulgare*	[20,22]	152.15	C_8_H_8_O_3_
2-Methylbutyraldehyde	96-17-3	98+	Adamas-beta	*S. bicolor*, *Z. mays*, *S. italica*	[23]	86.13	C_5_H_10_O
Benzaldehyde	100-52-7	99	Adamas-beta	*O. sativa*	[24,25]	106.12	C_7_H_6_O
Nonanal	124-19-6	95+	Tokyo Chemical Industry	*O. sativa*, *S. bicolor*, *A. hypogaea*, *S. italica*	[26,27]	142.24	C_9_H_18_O
n-Octanaldehyde	124-13-0	98	Adamas-beta	*O. sativa*, *A. hypogaea*,	[16,26]	128.21	C_8_H_16_O
3-Methylbutanal	590-86-3	98	Adamas-beta	*O. sativa*, *S. bicolor*, *Z. mays*, *S. italica*	[23]	86.13	C_5_H_10_O
Isobutyraldehyde	78-84-2	98+	Tokyo Chemical Industry	*O. sativa*, *S. bicolor*, *Z. mays*, *S. italica*	[23]	72.11	C_4_H_8_O
1-Pentanecarbaldehyde	66-25-1	98	Adamas-beta	*S. bicolor*, *O. sativa*, *A. hypogaea, S. italica*	[14,16,27]	100.16	C_6_H_12_O
6-Methyl-5-hepten-2-one	110-93-0	98	Adamas-beta	*O. sativa*	[28]	126.2	C_8_H_14_O
Acetone	67-64-1	98	Adamas-beta	*S. bicolor*	[14]	58.08	C_3_H_6_O
2-Pentanone	107-87-9	99	Adamas-beta	*O. sativa*	[21]	86.13	C_5_H_10_O
Palmitic acid	57-10-3	98+	Adamas-beta	*S. bicolor*, *S. italica*	[15]	256.42	C_16_H_32_O_2_
Linoleic acid	60-33-3	95+	Aladdin	*O. sativa*, *S. bicolor*, *S. italica*	[15,21]	280.45	C_18_H_32_O_2_
Acetic acid (glacial)	64-19-7	99	Adamas-beta	*Z. mays*	[29]	60.05	C_2_H_4_O_2_
Maltol (3-hydroxy-2-methyl-4-pyrone)	118-71-8	99	Adamas-beta	*T. aestivum*, *O. sativa*, *A. sativa*, *S. cereale*, *H. vulgare*	[20]	126.11	C_6_H_6_O_3_
Myrcene (stabilized with BHT)	123-35-3	75+	Adamas-beta	Storage grain	[19]	136.23	C_10_H_16_
Bis(2-methoxyethyl) phthalate	117-82-8	95	Adamas-beta	Storage grain	[19]	282.29	C_14_H_18_O_6_
Methanol	67-56-1	HPLC	Adamas-beta	-	-	32.04	CH_4_O
N-Phenyl-1-naphthylamine	90-30-2	99	Adamas-beta	-	-	219.28	C_16_H_13_N

Notes: *Sorghum bicolor (S. bicolor); Arachis hypogaea (A. hypogaea); Oryza sativa (O. sativa); Setaria italica (S. italica); Triticum aestivum (T. aestivum); Avena sativa (A. sativa); Secale cereal (S. cereale); Hordeum vulgare (H. vulgare); Zea mays (Z. mays)*.

**Table 2:** Primers used in this study.

Primer Name	Forward (5’–3’)	Reverse (5’–3’)	Product Length (bp)
For prokaryotic expression			
SzeaOBP1	CGGGATCCGGAACAAACGACCCCCAA (BamH1)	CGCTCGAGAAGATTGTGGTCCTCTTTAA (Xhol)	345
SzeaOBP28	CGGGATCCATGTTTGACGAATCCACATT (BamH1)	CGCTCGAGAACCATCATATAGGCTTCTG (Xhol)	372
For qRT-PCR			
SzeaOBP1	CGACTCCTTCTACCACCAC	AACACTGAACCGCATCTC	206
SzeaOBP28	AATCAAAGTGGAGCAA	TCAAAGCGTCAGGAAT	175
β-actin	GGGGCGAATACTGTGAGAAA	AGCAGGTTCAAAAGGCTCAA	192

**Table 3:** Binding affinities of the two SzeaOBPs with 27 tested ligands.

Ligand	SzeaOBP1		SzeaOP28	
	IC_50_ (μM)	Ki (μM)	IC_50_ (μM)	Ki (μM)
**Alkanes**				
Pentadecane	-	-	-	-
*N*-Hexadecane	-	-	-	-
**Alcohols**				
1-Nonanol	8.131 ± 0.274	6.944 ± 0.234	-	-
1-Hexanol	5.470 ± 0.186	4.671 ± 0.159	-	-
Capryl alcohol	7.814 ± 0.233	6.673 ± 0.199	-	-
Cetanol	3.218 ± 0.162	2.748 ± 0.139	-	-
**Aldehyde**				
Decanal	-	-	-	-
Valeraldehyde	5.715 ± 0.121	4.880 ± 0.103	-	-
Heptaldehyde	5.707 ± 0.130	4.874 ± 0.111	-	-
4-Hydroxy-3-methoxybenzaldehyde	4.368 ± 0.203	3.730 ± 0.173	24.680 ± 2.340	20.23 ± 1.918
2-Methylbutyraldehyde	4.916 ± 0.102	4.198 ± 0.087	-	-
Benzaldehyde	5.198 ± 0.092	4.439 ± 0.079	-	-
Nonanal	5.179 ± 0.111	4.423 ± 0.095	58.855 ± 1.342	48.242 ± 1.100
n-Octanaldehyde	3.769 ± 0.120	3.219 ± 0.103	-	-
3-Methylbutanal	7.617 ± 0.191	6.505 ± 0.163	-	-
Isobutyraldehyde	4.987 ± 0.162	4.259 ± 0.138	-	-
1-Pentanecarbaldehyde	6.067 ± 0.154	5.181 ± 0.132	59.42 ± 1.214	48.70 ± 0.852
**Ketones**				
6-Methyl-5-hepten-2-one	6.501 ± 0.150	5.552 ± 0.128	45.845 ± 1.232	37.578 ± 1.010
Acetone	5.320 ± 0.201	4.543 ± 0.172	48.555 ± 0.861	39.824 ± 0.731
2-Pentanone	11.62 ± 0.312	9.923 ± 0.267	-	-
**Acids**				
Palmitic Acid	28.28 ± 2.082	24.150 ± 1.778	49.746 ± 1.724	40.776 ± 1.414
Linoleic acid	-	-	-	-
Acetic acid (glacial)	18.43 ± 0.840	15.739 ± 0.717	-	-
**Others**				
Maltol (3-hydroxy-2-methyl-4-pyrone)	6.179 ± 0.390	5.277 ± 0.333	-	-
Myrcene (stabilized with BHT)	35.45 ± 0.931	30.27 ± 0.798	-	-
Bis(methylglycol) phthalate	4.762 ± 0.147	4.067 ± 0.125	38.865 ± 0.200	31.857 ± 0.164
(+)-a-Pinene	-	-	-	-

Notes: ‘-’ These compounds had no effective binding ability between ligand and protein.
